# Ultrasound Guided Intra-thymic Injection to Track Recent Thymic Emigrants and Investigate T Cell Development

**DOI:** 10.21769/BioProtoc.3017

**Published:** 2018-12-05

**Authors:** Haiguang Wang, David L. Owen, Lily J. Qian, Laura B. Chopp, Michael A. Farrar, Kristin A. Hogquist

**Affiliations:** Center for Immunology, University of Minnesota, Minneapolis, MN, USA; University of Pennsylvania Medical School, Philadelphia, PA, USA

**Keywords:** Ultrasound, Intra-thymic injection, Recent thymic emigrants, Thymus, T cell development, Invariant natural killer T (iNKT) cells, Regulatory T (Treg) cells

## Abstract

To track recent thymic emigrants (RTEs) or study T cell development in the thymus, intra-thymic injection of a cellular tag or precursor cells for various T cell lineages is often desired. However, the traditional surgical approach to expose the thymus for intra-thymic injection is time-consuming and can cause a high level of pain and stress to animals, which might disrupt immune homeostasis, potentially confounding the results. Here, we introduce an ultrasound guided intra-thymic injection procedure, which is non-surgical and minimally invasive to animals. This technique is relatively easy to learn and offers an efficient and accurate tool to track RTEs or perform intra-thymic transfer of various cell types to investigate their differentiation.

## Background

T cells play an essential role in protective immunity against invading pathogens and malignant self. The thymus is the site where T cells originate and develop; therefore, it has long been an organ of interest to study immune system development. Many of these studies involve tracking recent thymic emigrants (RTEs) or dissecting the differentiation steps of T cells into different lineages. The labeling of thymocytes via intra-thymic injection of a cellular tag like a biotinylating agent (NHS-biotin) or Fluorescein isothiocyanate (FITC), allows specific detection of RTEs in the periphery. Furthermore, it is often necessary to perform direct intra-thymic transfer of precursor cells to investigate precursor-product relationships during T cell development. As such, the ultrasound guided intra-thymic injection procedure described here provides a fast, easily learned and non-invasive technique to study such processes in the thymus.

 The traditional intra-thymic injection approach involves surgical procedures that cut the sternum to open the thoracic cavity to directly visualize and inject the thymus. Though this method has been used extensively, this technique is invasive and subjects animals to stress and possible mortality. Also, performing this surgical intra-thymic injection requires ample experience in mouse surgery. Furthermore, the efficiency and accuracy of this technique sometimes can be questionable, as the visualization of thymus is usually limited. To improve the efficiency, accuracy and success rate of intra-thymic injection, and reduce the stress of animals during the procedure, we describe here a non-surgical, minimally invasive ultrasound guided intra-thymic injection procedure. Using ultrasound imaging, the thymus can be clearly visualized without any surgical exposure, and accurate injection into the thymus can be further guided and monitored. We have successfully implemented this ultrasound guided intra-thymic injection approach in a variety of studies. As shown here and the original research papers (Wang and Hogquist, 2018; Owen *et al.*, 2018), we performed intra-thymic injection of biotin to label thymocytes and tracked the biotin^+^ RTEs in the periphery. Moreover, we were able to identify and sort precursor cells for invariant natural killer T (iNKT) cells and regulatory T (Treg) cells which, upon intra-thymic transfer into congenic hosts, substantially differentiated into all three iNKT effector subsets or mature Treg cells, respectively. Lastly, we also transferred purified wild-type (Wt) DN thymocytes into congenic recipients using ultrasound guided intra-thymic injection and detected robust development into CD4^+^ CD8^+^ double positive (DP), CD4^+^ single positive and CD8^+^ single positive thymocytes. Such a technique could be combined with genetic manipulation of DN thymocytes in order to identify cell-intrinsic factors important for T cell differentiation. In general, the ultrasound guided intra-thymic injection could serve as a convenient tool for further investigation of T cell emigration and development in the murine thymus.

## Materials and Reagents

1. 1.5 ml microcentrifuge tubes (DOT Scientific, catalog number: 509-FTG)
2. 15 ml conical centrifuge tubes (Corning, Falcon^®^, catalog number: 352097)
3. 70 μm cell strainers (Corning, Falcon^®^, catalog number: 352350)
4. 6-well plates (Corning, Costar^®^, catalog number: 3506)
5. 0.5 ml insulin syringes (EXELINT INTERNATIONAL, catalog number: 26028)
6. 3 ml syringes (Covidien, catalog number: 8881513918)
7. 5 ml FACS tubes with Cell-Strainer Cap (Corning, Falcon^®^, catalog number: 352235)
8. 96 round-bottom well plates (SARSTEDT, catalog number: 82.1582.001)
9. Aluminum foil (Spring Grove)
10. MACS LS columns (Miltenyi Biotec, catalog number: 130-042-401)
11. Facemask (3M, catalog number: 1820)
12. Medical tape (3M Transpore, catalog number: 1527-1; 3M Durapore, catalog number: 1538-1)
13. Paper towel
14. Alcohol pad (McKESSON, catalog number: 58-204)
15. Cotton pad (McKESSON, catalog number: 44082000)
16. B6 (C57BL/6NCr) mice (THE JACKSON LABORATORY, catalog number: 000664)
17. B6.SJL (B6-LY5.2/Cr) mice (THE JACKSON LABORATORY, catalog number: 002014)
18. *Tbx21* ^GFP^ KN2 BALB/cBYJ mice (have been previously described in Wang and Hogquist, 2018)
19. *Foxp3* ^GFP^ (B6.Cg-Foxp3tm2(EGFP)Tch/J) mice (THE JACKSON LABORATORY, catalog number: 006772)
20. CD45.1^+^ BALB/cBYJ (CByJ.SJL(B6)-Ptprca/J) (THE JACKSON LABORATORY, catalog number: 006584)
21. Eye ointment (MAJOR LubriFresh P.M., catalog number: 0904-6488-38)
22. Nair (Nair^TM^ HAIR REMOVER LOTION)
23. Ultrasound gel (aquasonic CLEAR^®^, catalog number: 03-08)
24. Phosphate buffered saline (PBS) (Corning, Mediatech, catalog number: 21-040-CV)
25. Isoflurane (Piramal Healthcare, catalog number: 001725CS)
26. Anti-Biotin MicroBeads (Miltenyi Biotec, catalog number: 130-105-637)
27. Viability dye Ghost Dye^TM^ Red 780 (TONBO Biosciences, catalog number: 13-0865-T100)
28. CD1d-tetramer (PBS57-loaded CD1d biotinylated monomers were from NIH tetramer core)
29. Streptavidin-PE (BD Biosciences, catalog number: 554061)
30. Streptavidin-BV421 (BioLegend, catalog number: 405225)
31. Anti-CCR7 (BD Biosciences, catalog number: 562675)
32. Anti-CD4 (BD Biosciences, catalog number: 563331)
33. Anti-CD8α (BD Biosciences, catalog number: 563786)
34. Anti-CD24 (BioLegend, catalog number: 101824)
35. Anti-NK1.1 (BioLegend, catalog number: 108718)
36. Anti-CD44 (TONBO Biosciences, catalog number: 80-0441-U025)
37. Anti-human CD2 (BioLegend, catalog number: 309218)
38. Anti-TCRβ (BD Biosciences, catalog number: 563221)
39. Anti-CD45.1 (BioLegend, catalog number: 110738)
40. Anti-CD45.2 (eBioscience, catalog number: 11-0454-81)
41. Anti-PLZF (BD Biosciences, catalog number: 563490)
42. Anti-ROR-γt (BD Biosciences, catalog number: 562684)
43. Anti-T-bet (BioLegend, catalog number: 644824)
44. Anti-CD25 (TONBO Biosciences, catalog number:65-0251-U100)
45. Anti-CD45.1 (BD Biosciences, catalog number: 563754)
46. Anti-CD90.2 (eBioscience, catalog number: 47-0902-82)
47. Anti-CD90.1 (eBioscience, catalog number: 57-0900-82)
48. Anti-CD73 (eBioscience, catalog number: 48-0731-82)
49. Sulfo-NHS-LC biotin (Thermo Fisher Scientific, catalog number: 21335)
50. Fetal bovine serum (FBS) (Atlanta Biologicals, catalog number: S11150), heat inactivated at 65 °C
51. Ethylenediaminetetraacetate acid (EDTA) (Fisher Scientific, catalog number: BP120-500)
52. Sodium azide (Fisher Scientific, catalog number: BP922I-500)
53. Ammonium chloride (NH_4_Cl) (Sigma-Aldrich, catalog number: A4514-500G)
54. Potassium bicarbonate (KHCO_3_) (Fisher Scientific, catalog number: P235-500)
55. Bovine serum albumin (BSA) (Sigma-Aldrich, catalog number: A7906)
56. autoMACS Rinsing Solution (Miltenyi Biotec, catalog number: 130-091-222)
57. MACS BSA Stock Solution (Miltenyi Biotec, catalog number: 130-091-376)
58. Betadine (BETADINE Surgical Scrub, catalog number: 67618-151-17)
59. FACS buffer (see Recipes)
60. ACK lysis buffer (see Recipes)
61. MACS buffer (see Recipes)

## Equipment

1. Class-II biosafety cabinet/laminar flow hood
2. Vevo^®^ 2100 Imaging System (Visual Sonics, see Figure 1A)
3. Acrylic chamber (see Figure 1B)
4. MACS Multistand (Miltenyi Biotec, catalog number: 130-042-303)
5. QuadroMACS Separator (Miltenyi Biotec, catalog number: 130-090-976)
6. Benchtop centrifuge (Beckman Coulter, model: Allegra X-12-R)
7. Hemacytometer (Sigma-Aldrich, catalog number: Z359629-1EA)
8. BD Fortessa H0081 Flow cytometer
9. BD FACSAria II Cell Sorter
10. MS550 transducer (Visual Sonics, see Figure 1A)
11. Refrigerator (2-8 °C; Isotemp LABORATORY REFRIGIRATOR)
12. isoflurane vaporizer
13. heating pad

## Software

1. FlowJo 10.4.0 (https://www.flowjo.com/)

## Procedure

1. Ultrasound guided intra-thymic injection
2. Pick up one mouse and place in the acrylic chamber connected to an isoflurane vaporizer (Figures 1A and 1B). We normally use the mouse ranges from 6 to 10 weeks of age.
3. Induce anesthesia using the acrylic chamber with 3% of isoflurane in O_2 _administered at a rate of 600 ml/min into the chamber (Figure 1B).
4. Frequently check the anesthesia state of the mouse; the mouse should be anesthetized within 3 min.
5. Take mouse out of the chamber and quickly transfer to the immobilization heating pad, cover the mouse nose with a facemask to maintain anesthesia with 3% of isoflurane in O_2_ administered at a rate of 600 ml/min (Figure 1C).
6. Apply eye ointment on both eyes to prevent them from drying during anesthesia period.
7. Flip the mouse belly up and immobilize limbs with medical tape, during which the mouse should be maintained under anesthesia with the facemask (Figure 1C).
8. Apply Nair on chest area, wait for 1-2 min to effect, remove hair with a paper towel. Swipe the chest area with betadine and clean up with an alcohol pad to sterilize the area.
9. Apply ultrasound gel on the chest area and then perform the ultrasound scan using the Vevo^®^ 2100 Imaging System (Visual Sonics) with the MS550 transducer, which ranges from 32 to 40 MHz. Fix the transducer right above the chest area in direct contact with ultrasound gel; the live ultrasound imaging should be as shown, while the syringe will approach the thymus from the side of chest (Figures 1C and 1D).
10. Identify the thymus in the ultrasound image as shown in Figure 1D.
11. Fill the syringe (29G1/2 needle) with 10-20 µl of NHS-biotin at 1 mg/ml in PBS or with cell suspension, tightly fix the syringe in the syringe stand on the right side of the animal and adjust to approximate 135° angle point from the ground (Figure 1C).
12. First, approach the needle into the gel between mouse and transducer until clearly visualized in the ultrasound imaging as a high-density white dot (Figure 1E). Then, slide the syringe up and down to target the needle directly above the thymus (Figure 1E).
13. Pull the needle out of the gel, lower the syringe (till the needle tip points to the chest area), advance the needle into the mouse through the ribs and continuously penetrate toward the thymus. Frequently monitor the ultrasound image until the needle tip is visualized within the area of thymus (Figure 1F), perform injection, then pull the needle out of the mouse.
14. After injection, remove the ultrasound gel using a cotton pad and wipe the chest area clean with an alcohol pad. Remove medical tape restraints on mouse limbs, remove mouse from nose cone and place the mouse back in the cage for recovery. The mouse should recover in 2-3 min and behave similar to before injection.
  **Figure 1.**
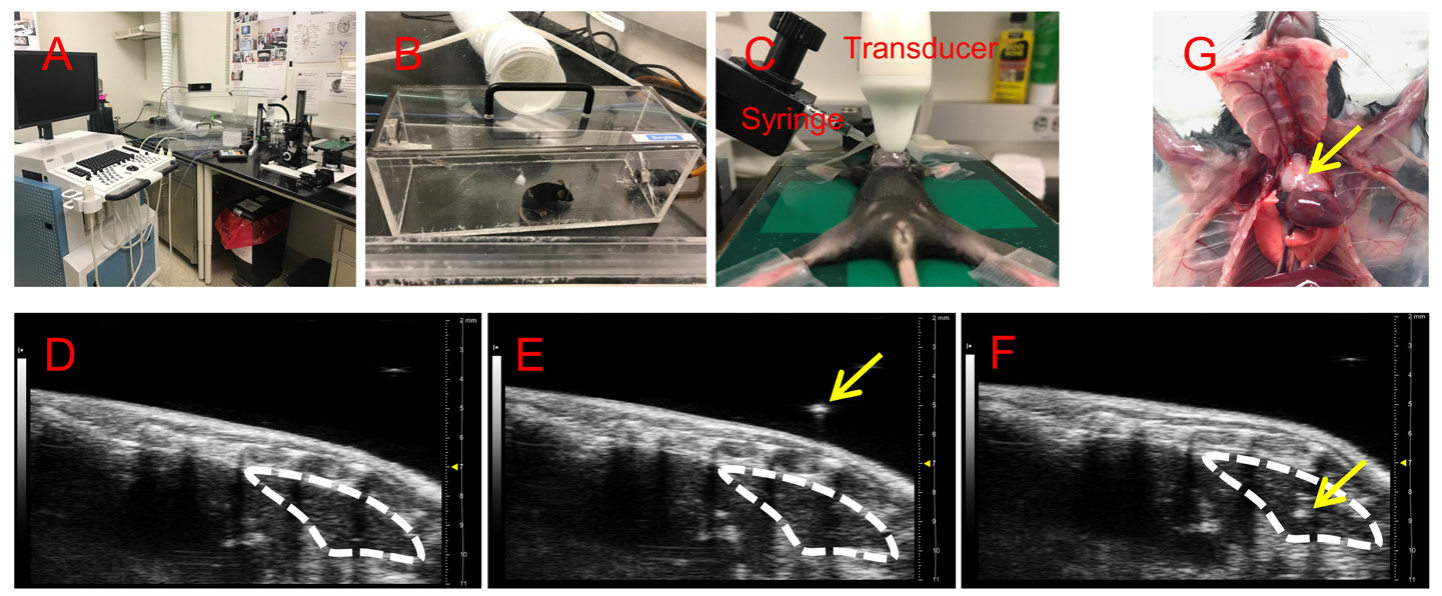
Ultrasound imaging system and ultrasound guided intra-thymic injection. A. Vevo^®^ 2100 Imaging System for ultrasound imaging of mouse, including the acrylic chamber and the immobilization heating pad. B. Mouse in the acrylic chamber for anesthesia. C. Mouse immobilized on the heating pad for ultrasound imaging and intra-thymic injection. D. The ultrasound imaging of mouse chest area before injection, the white dashed line outlines the thymus. E. The white dashed line outlines the thymus; the yellow arrow indicates the needle tip in the gel right above the thymus. F. The white dashed line outlines the thymus; the yellow arrow indicates the needle penetrating inside the thymus during injection. G. Image for the localization of thymus in the chest cavity of mouse. The yellow arrow indicates the thymus.
15. Track recent thymic emigrants (RTEs) in the periphery
16. To track RTEs, perform intra-thymic injection of NHS-biotin as described in Procedure A.
17. Euthanize the mouse 24-72 h later, collect the thymus (Figure 1G) in ice cold FACS buffer and spleen in ice cold MACS buffer.
18. Prepare single cell suspension from the thymus and spleen by mashing the tissues with the plunger of a 3 ml syringe and passing cell suspension through a 70 μm cell strainer.
19. Determine the cell count of each sample using hemacytometer.
20. In accordance with the Miltenyi protocol, resuspend spleen cells in 80 μl of MACS buffer per 10^7^ cells, add 20 μl of Anti-Biotin MicroBeads per 10^7^ cells. Vortex to mix well and incubate for 45 min in the refrigerator (2-8 °C).
21. Wash cells with 2 ml of MACS buffer per 10^7^ cells and centrifuge to pellet at 300 *x g* for 10 min. Aspirate supernatant.
22. After wash, resuspend cell pellet with 500 μl of MACS buffer up to 10^8^ cells.
23. Proceed to the magnetic enrichment of biotin^+^ cells using LS columns according to the Miltenyi protocol.
24. Collect the flowthrough and bound fraction of cells and perform flow cytometry to identify biotin^+^ cells through staining with streptavidin-PE or streptavidin-BV421.
25. Developmental outcome of transferred progenitors
26. Perform intra-thymic injection of sorted or purified cells of choice into congenically distinct recipient mice as described in Procedure A to study T cell development in the thymus. In our hands, we successfully transferred invariant natural killer T (iNKT) progenitor cells, T regulatory (Treg) progenitor cells and DN thymocytes, and monitored their differentiation into iNKT effector subsets, mature Treg cells, and mature SP thymocytes, respectively.
27. Briefly, sort the iNKT progenitors from thymocytes of T-betGFP/KN2 mice, Treg progenitors from thymocytes of *Foxp3* ^GFP^ mice with a FACS sorter, or purify and enrich the DN thymocytes using LS column by depleting CD4^+^, CD8^+^ and CD4^+^ CD8^+^ thymocytes of B6 mice. More details concerning the background of iNKT cell and Treg cell development, as well as gating and sorting strategies of their precursors were shown in the original research paper (Wang and Hogquist, 2018; Owen *et al.*, 2018).
28. Wash the sorted or purified cells 2 times with 10 ml of PBS and centrifuge to pellet at 300 *x g* for 10 min. Aspirate supernatant.
29. After the second wash, resuspend cell pellet in about 20 μl of PBS, keep cell suspension on ice before injection.
30. Perform the intra-thymic injection of the cell suspension as described in Procedure A.
31. Euthanize the mouse after the injection (experimental end points should be determined empirically for each study), collect the thymus in ice cold FACS buffer.
32. Prepare single cell suspension from the thymus by mashing the tissues with the plunger of a 3 ml syringe and passing cell suspension through a 70 μm cell strainer.
33. Perform flow cytometry to identify the transferred cells through staining of the congenic markers (CD45.1 and CD45.2) and/or other related cell markers.

## Data analysis

1. The analysis of flow cytometry data was performed using Flowjo 10.
2. Intra-thymic injection of NHS-biotin provided robust labeling of nearly half of the total thymocytes (Figure 2A), more results were shown in Figure 1–figure supplement 3 of the original research paper (Wang and Hogquist, 2018).
3. To track the RTEs in the periphery, mice were euthanized, and spleens were collected for analysis 24 h after intra-thymic injection of biotin. RTEs were identified as biotin^+^ cells in the periphery. Since RTEs were rare events in the peripheral T cells, magnetic enrichment of biotin^+^ cells was performed (see Procedure B for detailed protocol), and this approach provided clear detection of biotin^+^ cells to track RTEs (Figure 2B). More results were shown in Figure 1–figure supplement 3 and Figures 2A-2B of the original research paper (Wang and Hogquist, 2018).
4.
  **Figure 2.**
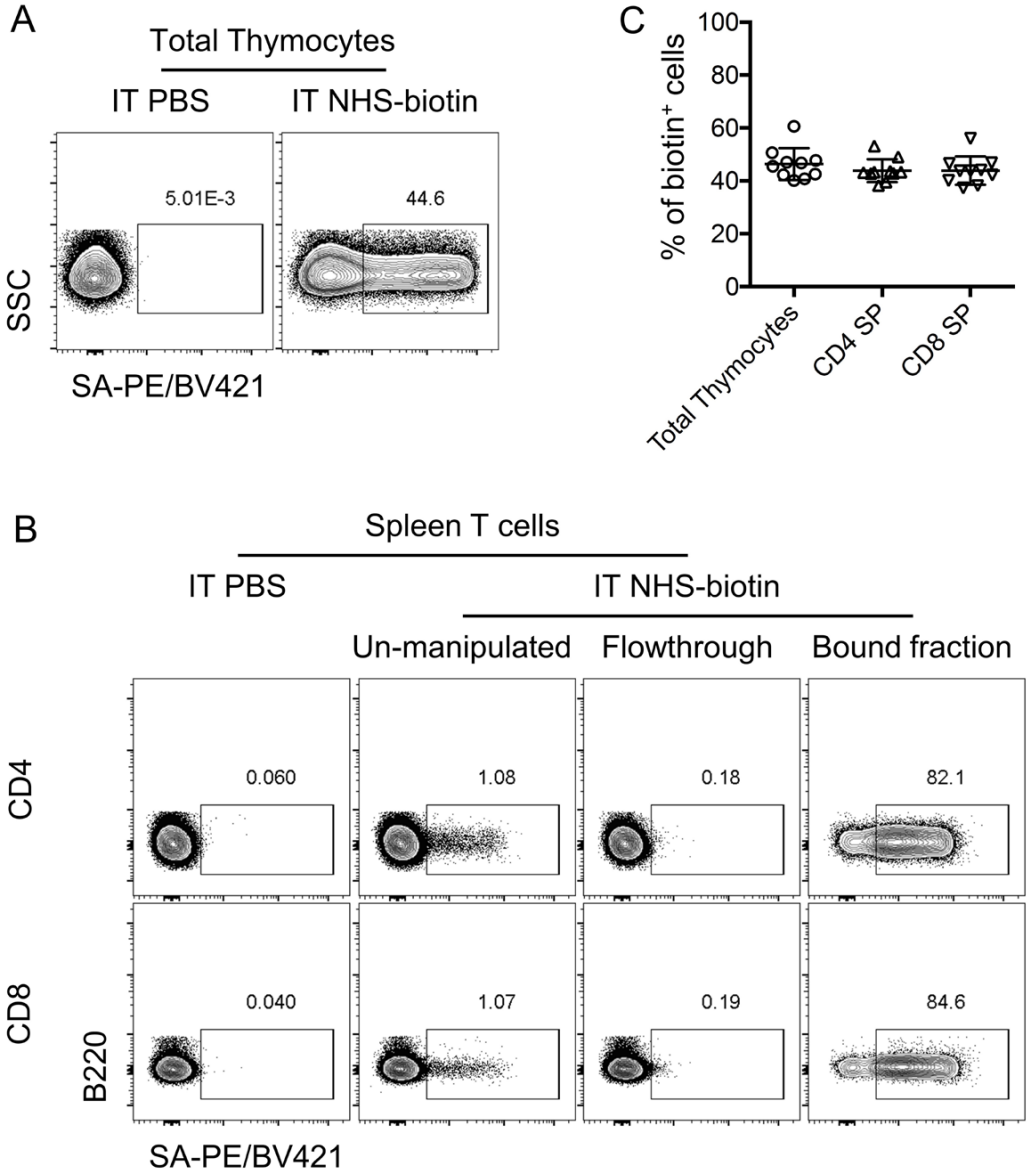
Ultrasound guided intra-thymic injection of biotin to track RTEs. A. Representative flow cytometry plots of biotinylated cells revealed by streptavidin-BV421 staining in total thymocytes 24 h later after intra-thymic injection of PBS (left column) or NHS-biotin (right column). B. twenty-four hours after the intra-thymic injection of NHS-biotin, biotin^+^ RTEs could be specifically identified in the spleen CD4^+^ (upper row) or CD8^+^ (bottom row) T cells populations using magnetic enrichment. C. Frequency of biotin^+^ cells revealed by streptavidin-PE/BV421 staining in CD4 SP, CD8 SP and total thymocytes in the thymus 24 h after intra-thymic injection of NHS-biotin.
  iNKT progenitors were intra-thymically transferred into congenic distinct recipients, and 5 days later, a substantial differentiation into all three iNKT effector subsets was detected. More results were shown in Figures 1E and 1F of original research paper (Wang and Hogquist, 2018).
5. Immature Treg progenitors were intra-thymically transferred into congenic distinct recipients, and 6 days later, significant differentiation into CD25^+^ Foxp3^+^ mature Tregs was detected. More results were shown in Figure 1 of original research paper (Owen *et al.*, 2018).
6. DN thymocytes were purified and enriched using LS column (Figure 3A) and were intra-thymically transferred into congenic distinct recipients (Figure 3B). The recipient mice were irradiated sub-lethally (500 Rads) the day before receiving intra-thymic injection. 14 days later, the transferred cells could be readily identified as CD45.1^+^ cells, and a substantial differentiation into DP thymocytes, CD4^+^, and CD8^+^ thymocytes was detected (Figure 3B).
  **Figure 3.**
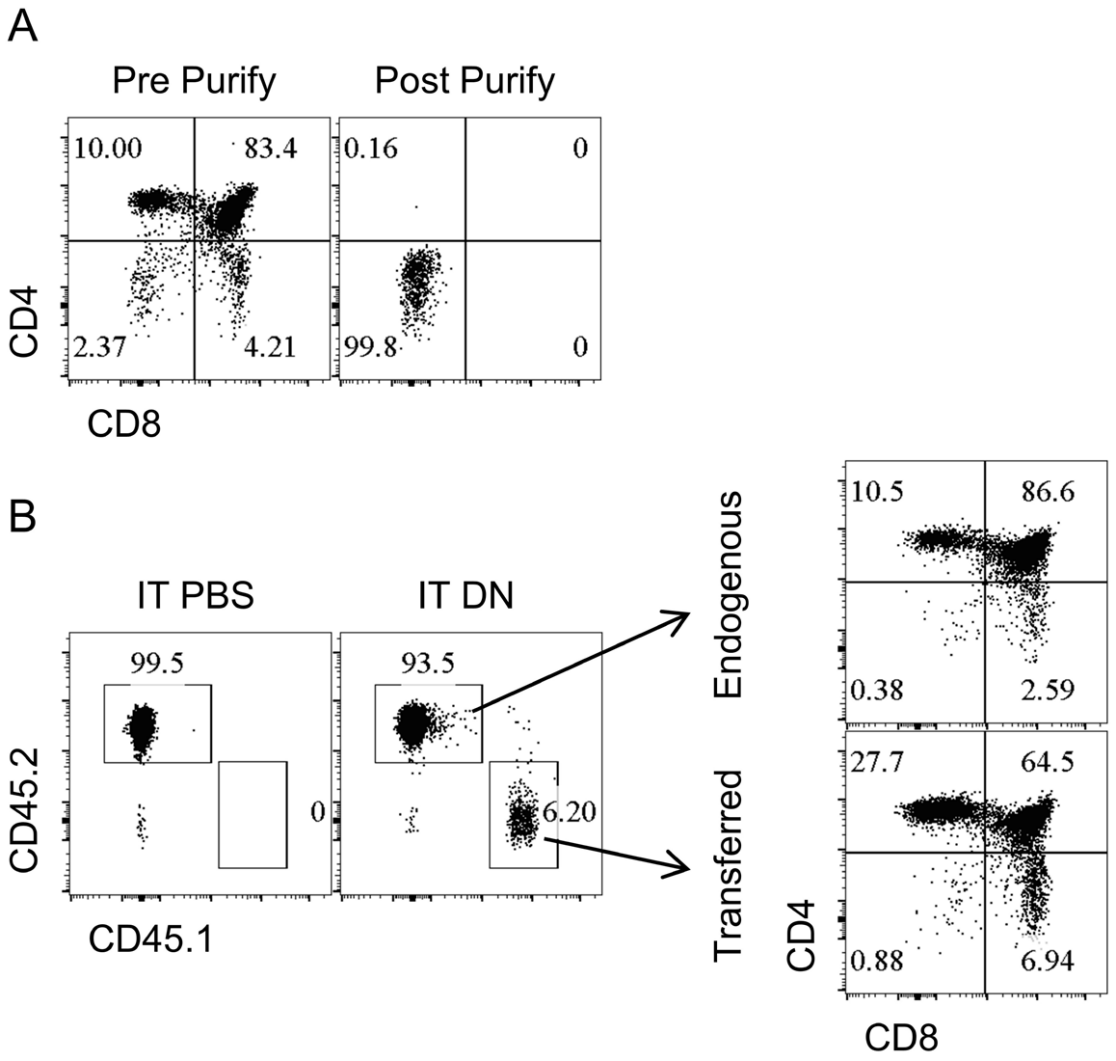
Intra-thymic transfer of DN thymocytes for investigation of T cells development. A. CD4 and CD8 expression before (left column) and after (right column) the purification of DN thymocytes. B. The purified CD45.1^+^ DN thymocytes or PBS were intra-thymically transferred in CD45.2^+^ congenic recipients and transferred cells could be specifically detected according to their expression of CD45.1 and CD45.2 (left two columns); 14 days after intra-thymic transfer, the DN thymocytes showed robust development into DP thymocytes, CD4^+^ and CD8^+^ thymocytes (right bottom row) similar to the endogenous thymocytes (right upper row).

## Recipes

1. FACS buffer (store at 4 °C, ~10 ml for each sample)
  2% FBS
  0.02% sodium azide
  500 ml PBS
2. ACK lysis buffer (store at 4 °C, ~5 ml for each sample)
  150 mM NH_4_Cl
  10 mM KHCO_3 _
  0.1 mM EDTA
  1 L ddH_2_O
  Adjust pH to 7.2-7.4
3. MACS buffer (store at 4 °C, ~30 ml for each sample)
  1,450 ml autoMACS Rinsing Solution
  75 ml MACS BSA Stock Solution
